# TiN nanoparticles: small size-selected fabrication and their quantum size effect

**DOI:** 10.1186/1556-276X-7-80

**Published:** 2012-01-18

**Authors:** Luis Carlos Hernández Mainet, Luis Ponce Cabrera, Eugenio Rodriguez, Abel Fundora Cruz, Guillermo Santana, Jorge Luis Menchaca, Eduardo Pérez-Tijerina

**Affiliations:** 1 Laboratory of Laser Technology, CICATA-IPN, Altamira, Tamaulipas, 89600, Mexico; 2 Instituto de Ciencia y Tecnología de Materiales (IMRE), Universidad de La Habana, La Habana, 10400, Cuba; 3 Instituto de Investigaciones en Materiales, Universidad Nacional Autónoma de México, CP 04510, Coyoacán, México; 4 Centro de Innovación, Investigación y Desarrollo en Ingeniería y Tecnología (CIIDIT), Universidad Autónoma de Nuevo León, Monterrey, Nuevo León, CP 66450, México

## Abstract

Size-selected TiN nanoclusters in the range of 4 to 20 nm have been produced by an ionized cluster beam, which combines a glow-discharge sputtering with an inert gas condensation technique. With this method, by controlling the experimental conditions, it was possible to produce nanoparticles with a high control in size. The size distribution of TiN nanoparticles was determined before deposition by mass spectroscopy and confirmed by atomic force microscopy. The size distribution was also analyzed using a high-resolution transmission electron micrograph. The photoluminescence [PL] spectra of TiN nanoparticles at different sizes were also experimentally investigated. We reported, for the first time, the strong visible luminescence of TiN nanoparticles on Si (111) wafer due to the reduced size. We also discussed the PL intensity as a function of the nanoparticle size distribution.

## Introduction

Metal nanoparticles 1 to 100 nm in size and 10^2 ^to 10^8 ^atom aggregates (known as clusters) have demonstrated different physical-chemical properties from their bulk. The reasons for these properties can be attributed to the large portion of surface atoms and quantum size effect, which is caused by the reduced size in three dimensions. When the cluster is very small, the number of atoms at surfaces or grain boundaries is comparable to the number of atoms in the crystalline lattice. Also, with the decrease of the cluster size, the electronic properties start to change. This effect is called the 'quantum confinement effect', which can be observed as a shift in the optical bandgap or exciton energy depending on the nanoparticle diameter. Several investigations have been carried out to study the particle size effects on their physical-chemical properties. A typical example is that the melting temperature of nanoparticles strongly depends on the size and shape and is substantially lower than the bulk melting temperature [1-3]. For nanoscience, the study of size-selected nanocluster is very important to understand the fundamentals of solid physics in a nanometric scale because it is the bridge in the gap between individual atoms and condensed matter.

Metal nanoparticles are used in a broad spectrum of applications such as in biomedicine [4], optoelectronics [5], solar cells [6], and anti-wear coatings [7]. Nowadays, they are involved in many products and applied in several technologies. Most metal nanoparticles' production processes require a precise control of narrow range size. In particular, especial conditions are necessary to produce very small size-selected nanoparticles at an industrial scale [8]. From a technological viewpoint, metal clusters can be regarded as the precursors to a new generation of nanostructured materials and devices. The fabrication of nanoparticles by controlling their size and shape is one of the challenging tasks for nanotechnology.

Among different techniques to obtain nanoparticles, ionized cluster beam deposition [ICBD] has been receiving great attention due to its control of size-selected nanoparticles [9]. The novel technique combines plasma sputtering and gas aggregation to produce nanoclusters from a few atoms to a few thousand atoms. The general setup of this system includes magnetron sputtering, a cluster aggregation zone, a mass filter, and a deposition chamber. Using a magnetron discharge, hot atoms are generated by Ar^+ ^bombardment on the target surface. The atoms are cooled and condensed in a cold inert gas to create the clusters. The cluster size can be controlled by adjusting the sputter yield, gas pressure, volume of the cluster growth region, and bias voltage. A mass filter located along the central axis of the system allows for selection of the cluster size. The clusters are accelerated toward the substrate surface by a bias voltage application. Finally, clusters join together, whether during the flight to the substrate or at the target surface, to form the nanoparticles.

Titanium nitride [TiN] has been generally applied in industrial coatings with high demands on hardness and adhesion as well as high thermal stability and good conductivity [10]. Due to this important feature, TiN has been widely used as a hard and protective coating for cutting tools or in electronic devices. Based on the properties of the bulk materials, TiN nanoparticles are being used as an additive element in protective coatings to enhance the adhesion properties [11] and a catalyst support material for noble metals for application in PEM fuel cells [12]. Several techniques, both chemical vapor deposition and physical vapor deposition, have been used to deposit TiN coatings. However, the industrial production of TiN nanoparticles is still beginning to take its first steps. ICBD is one technique that can be applied to produce TiN nanoparticles [13-16]. The investigation of TiN nanocluster deposition by ICBD can help to improve the production of nanomaterials and to understand their physicochemical properties at a nanoscale.

The fabrication of nanoparticles is an elaborate procedure. The following characterization is a complex but important task. Atomic force microscopy [AFM] and high-resolution transmission electron microscopy [HRTEM] have demonstrated to be powerful techniques to determinate the size of nanoparticles below 10 nm [17,18]. In addition, photoluminescence [PL] spectroscopy has emerged as an important tool for studying the luminescence of nanoparticles. The origin of such luminescence is often associated to quantum confinement effects in which the position of the PL energy peak depends fundamentally on the nanoparticle size.

In this paper, TiN nanoparticles with a narrow-sized range are deposited on Si (111) substrates at room temperature by ICBD method. The size distribution of the TiN clusters is measured before deposition by mass spectrometry and after by transmission electron microscopy [TEM] and AFM techniques. The crystalline structure of the TiN nanoparticles is further confirmed by the micrograph fast Fourier transform [FFT] analysis. A statistical detailed analysis of the TiN nanoparticle size distribution as a function of bias voltage is performed by HRTEM. The PL spectra of TiN nanoparticles at different sizes are also investigated experimentally. We reported for the first time, the strong visible luminescence of TiN nanoparticles on Si (111) wafer due to the reduced size.

## Experimental details

### Cluster production and co-deposition

**Figure 1:**
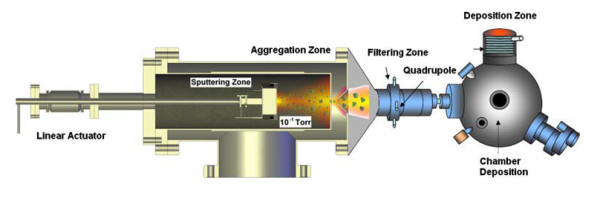
**Scheme of the nanocluster system deposition**.

A NanoSys 500 deposition system, built by Mantis Deposition Ltd (Thame, Oxon, UK), was used to produce size-selected TiN nanoclusters [19]. Figure 1 shows a schematic diagram of the experimental setup. The fabrication of noble metal nanoclusters using Nanosys 500 has been described elsewhere by other authors [18]. The difference in producing TiN clusters is the use of a reactive gas (N_2_) aggregation process.

**Figure 2:**
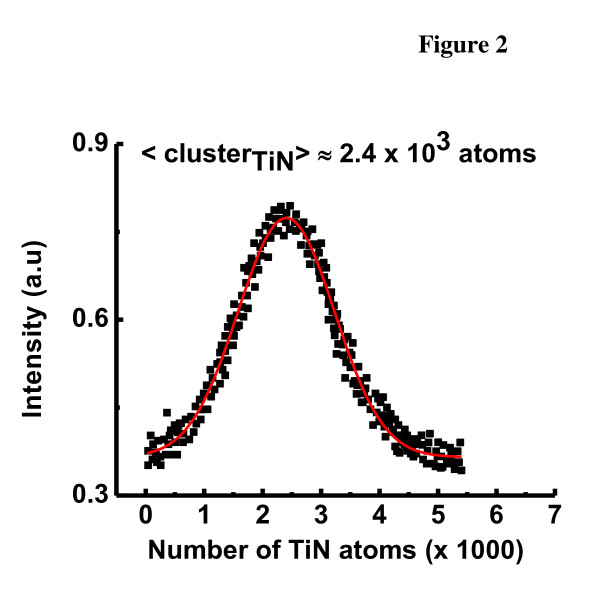
**Mass spectrum of (TiN)^+^_n _clusters**.

Inside the cluster generation zone, the TiN clusters were produced by sputtering a Ti target in an inert gas atmosphere of Ar^+ ^and by adding directly over the sputter target a small flux of nitrogen gas (20 sccm). When the Ti target is sputtered, the clusters are formed by multiple collisions between the high-density particles (sputtered particles and also argon and nitrogen gasses). During cluster formation, the sputter chamber is kept at a low temperature by a coolant mixture (2°C), and the pressure was set at 1 × 10^-4 ^Torr. The residence time within the aggregation zone can be diversified by varying the length of the aggregation region with the linear motion drive. The cluster size can be controlled by varying the principal parameters: the sputter power, flow of gasses, and the aggregate zone length (variable using a linear drive). The clusters flow together with the argon gas through a variable orifice towards a mass filter. The mass distribution is monitored *in situ *by a MesoQ mass filter (Mantis Deposition LTD, Thame, Oxon, UK) before the deposition. This mass filter has been specifically added for the purpose of high-resolution measurement and filtering of nanoclusters between 1 × 10^3 ^and 3 × 10^7 ^amu. A typical mass distribution spectrum of the obtained TiN^+ ^clusters is presented in Figure 2. The mean cluster diameter was estimated from the cluster masses by using the specific density and the molar volume of the TiN.

Once the size clusters are selected by the MesoQ mass filter, they are accelerated by applying a bias voltage (*V*_b_) to a substrate in a high vacuum with a base pressure of 10^-8 ^Torr. The nanoparticles were deposited onto silicon wafer. The substrates were cleaned in successive ultrasonic baths of acetone and isopropyl alcohol. The depositions were performed at room temperature without any heating and were applied different bias voltages (3 and 6 kV). The nanoparticle size was controlled by regulating the magnetron power, gas flow (Ar and N_2_), and aggregation zone length. These parameters were varied to produce particles of different sizes onto the substrate.

### Characterization of TiN nanoparticles

The size distribution and morphological characterization were performed by AFM analysis, using a (Veeco Instruments Inc., Plainview, NY, USA) multimode scanning probe microscope in hard tapping mode. The sample surface was scanned at 1 Hz and within 1 μm^2^. The Image Processing and Data Analysis software (Version 2.1.15) by TM Microscope (Camarillo, CA, USA) was used for image analysis.

For TEM characterizations, TiN nanoparticles were grown on copper grids. Samples were characterized by HRTEM using a 300-kV FEI Titan 80-300 STEM/TEM microscope (FEI Co., Hillsboro, OR, USA). The HRTEM images were used to study the TiN crystalline structure by micrograph FFT analysis. A detailed statistical analysis of TiN nanoparticles after deposition was performed by measuring several hundreds of nanoparticles using the HRTEM micrographs. The size distribution, the nearest neighbor distance (*d*_NN_), and the covered area on the surface were extracted from these calculations. The procedure was carried out by manually outlining the particles from several dozens of low- and high-resolution TEM images. Once digitized and saved in the proper format, the image was processed using the Gatan Digital Micrograph and Mathematical software (Gatan, Inc., Pleasanton, CA, USA).

PL studies were carried out at room temperature in a conventional PL system. An He-Cd laser (*λ *= 325 nm at 16 mW) was employed as the excitation source. The outgoing radiation from the sample was focused on the entrance slit of a 50-cm Acton monochromator (Princeton Instruments, Trenton, NJ, USA). The detection was carried out using a Princeton Instrument photomultiplier tube to a photon counter. All the spectra were corrected for the spectral response of the system.

## Results

### Size distribution of TiN nanoparticles

**Figure 3:**
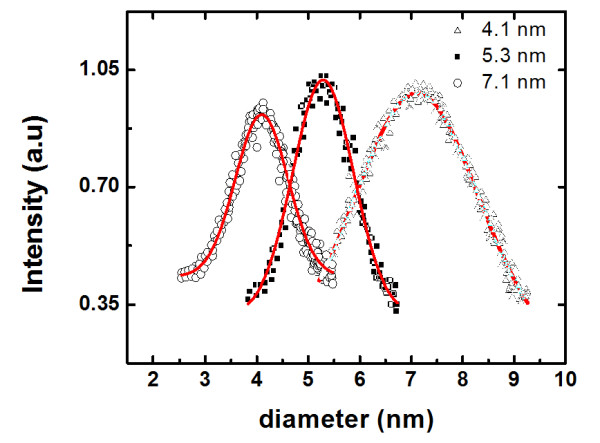
**Size distribution of TiN nanoclusters recorded by a MesoQ mass filter at 4.1, 5.3, and 7.1 nm**.

Figure 3 displays the typical size distribution recorded by the mass filter for three samples containing nanoparticles with different diameters. The mean nanocluster diameter was obtained after fitting a Gaussian function to the experimental data (Figure 3, solid lines). The figure displays the nanoclusters having a narrow size distribution of 4.1, 5.3, and 7.1 nm with a full width at half maximum of 1.2, 1.4, and 2.1 nm, respectively. Moreover, a good separation of peaks is clearly observed demonstrating that the mass filter can resolve the nanoparticle diameter with a difference at approximately 1 nm. Hence, the filtering process allows the selection of particles with a high resolution in size higher than 1 nm, which could be used in either scientific research or technological development. Varying the critical parameters on the system (gas flow, partial gas pressure, magnetron power, aggregation zone length), we were able to produce small nanoparticles at different sizes: 4.1 ± 0.2, 5.3 ± 0.4, 6.2 ± 0.2, 7.1 ± 0.3, 14.9 ± 0.7, and 20.3 ± 0.8 nm.

**Figure 4:**
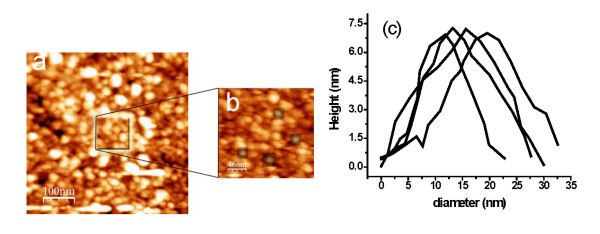
**AFM images of TiN nanoparticles**. (**a**) Aggregation of clusters due to coagulation of neighboring clusters (time of deposition is 360 s), (**b**) magnification, and (**c**) height profile of 7.1 nm.

After the deposition of TiN nanoparticles on Si (111) wafer, the morphology and size distribution were analyzed using the AFM images. Figure 4 shows a typical AFM image of TiN nanoparticles. From the figure, it is possible to observe the aggregation of nanoparticles, which could be explained due to clusters landing on top of each other before the layer is completed. For longer deposition times, it is expected that even more clusters get aggregated. The magnification in Figure 4a, b was used to measure the height profiles of four nanoparticles (Figure 4c). The height shows a roughly Gaussian profile, which allows to consider the nanoparticle as quasi-spherical.

**Figure 5:**
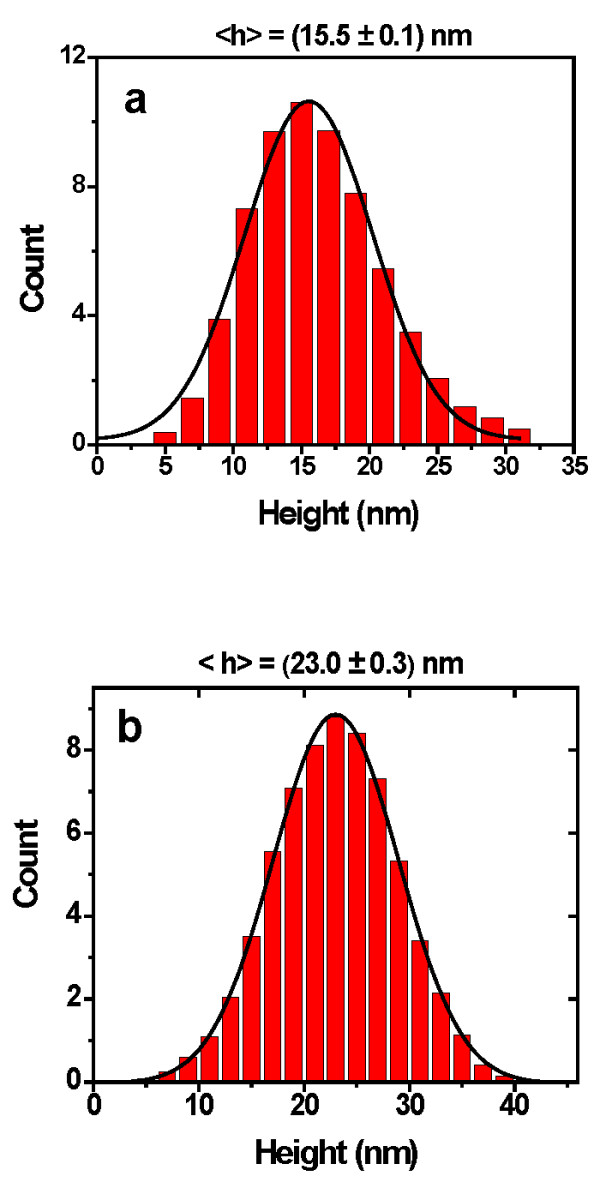
**AFM height histograms for TiN nanoparticles filtered by MesoQ**.

AFM height histograms for TiN nanoparticles at different filtered sizes are shown in Figure 5. These histograms show narrow height distributions with averages of 15.5 nm (Figure 5a) and 23.0 nm (Figure 5b). Usually in an AFM measurement, the distance in the *z*-axis is directly related to the nanoparticle size. However, in the *xy *plane, the distance is enlarged regarding the real spatial dimensions because the width of the AFM tip is distorted by the combination of the nanoparticle shape and tip geometry. In this case, the widths of height histograms do not refer to the real sizes of nanoparticles. Therefore, the results of size distribution for both mass filter and AFM height profiles support the effective mass filter to control the TiN nanoparticle size.

**Figure 6:**
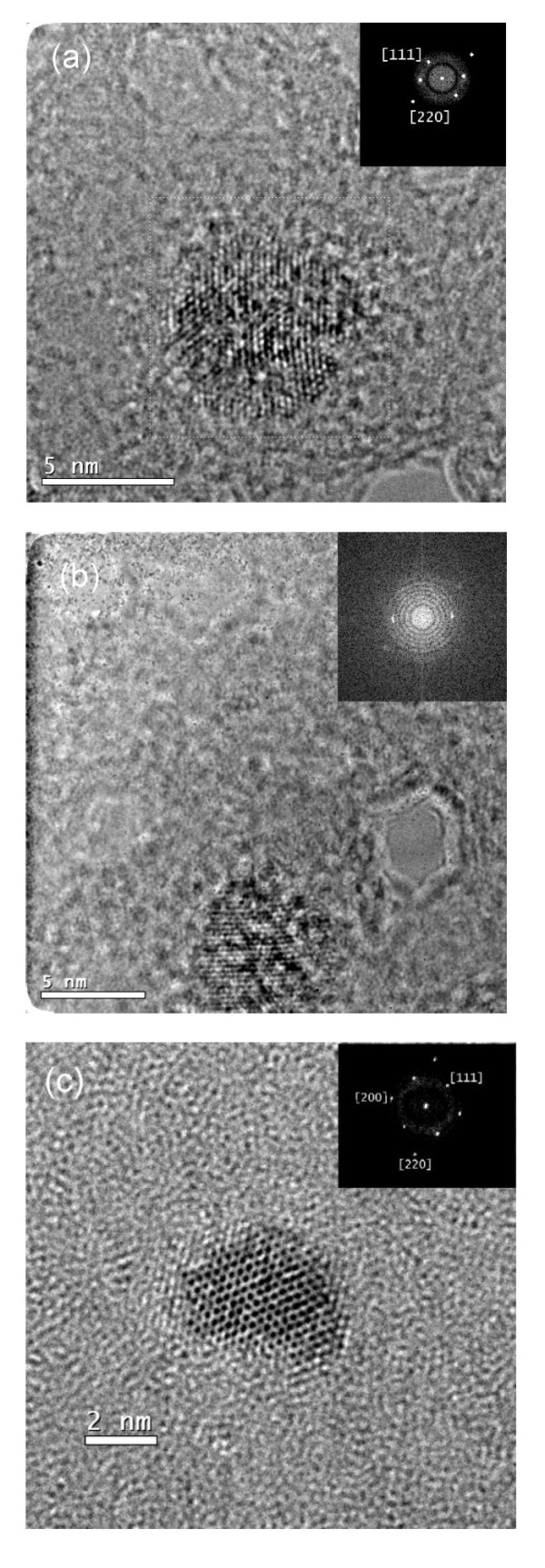
**HRTEM micrographs and the corresponding FFT of TiN nanoparticles grown by ICBD technique**. (**a**) 7.1 nm, (**b**) 6.2 nm, and (**c**) 4.1 nm. Inset: the corresponding FFT showing the [111, 200], and [220] directions of the TiN FCC structure.

The nanoparticle size was directly measured by HRTEM micrograph. Figure 6 shows TiN nanoparticles with sizes of 7.1 (Figure 6a), 6.2 (Figure 6b), and 4.1 nm (Figure 6c), where the two-dimensional atom arrangement and circular shape can be observed. The nanoparticle size was directly measured from the HRTEM micrographs using suitable tools of the image processing software. The crystalline structure of the TiN nanoparticles was further confirmed by FFT analysis (insert in the figures). The FFT shows a diffraction pattern corresponding to planes of the TiN face-centered cubic [FCC] structure with a lattice parameter of 4.2417 Ǻ [20]: (111) [*d*_111 _= 2.529 Å], (200) [*d*_200 _= 2.134 Ǻ], and (220) [*d*_220 _= 1.561 Å]. According to the AFM height profiles and plan-view TEM image, we can strongly consider the shape of nanoparticles as quasi-spherical.

**Figure 7:**
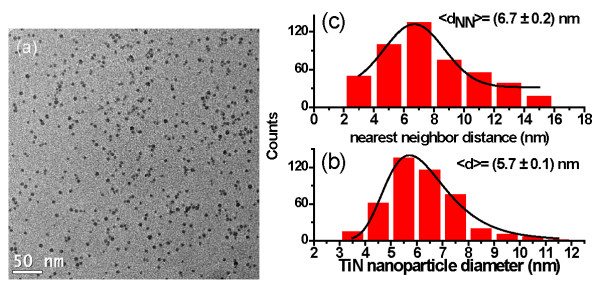
**Bias voltage, size distribution, and nearest neighbor distance**. (**a**) HRTEM micrograph of TiN nanoparticles grown at 3 kV bias voltage, (**b**) the distribution of size, and (**c**) the nearest neighbor distance.

**Figure 8:**
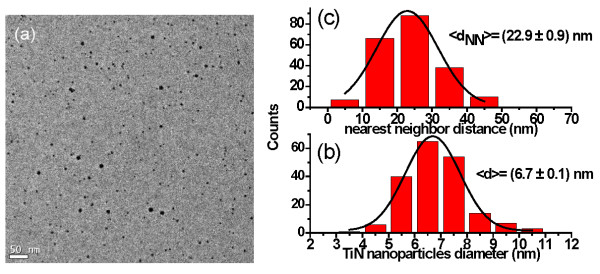
**Bias voltage, size distribution, and nearest neighbor distance**. (**a**) HRTEM micrograph of TiN nanoparticles grown at 6 kV bias voltage, (**b**) the distribution of size, and (**c**) the nearest neighbor distance.

The size distribution, cover surface, and nearest neighbor distance were also statistically analyzed as a function of the bias voltage. TiN nanoparticles produced at 3 and 6 kV bias voltage on TEM grids are shown in Figures 7a and 8a, respectively. The covered surface by the nanoparticles at 3 kV is around 8.2%, while at 6 kV, they cover only 2.1% of the surface.

**Figure 9:**
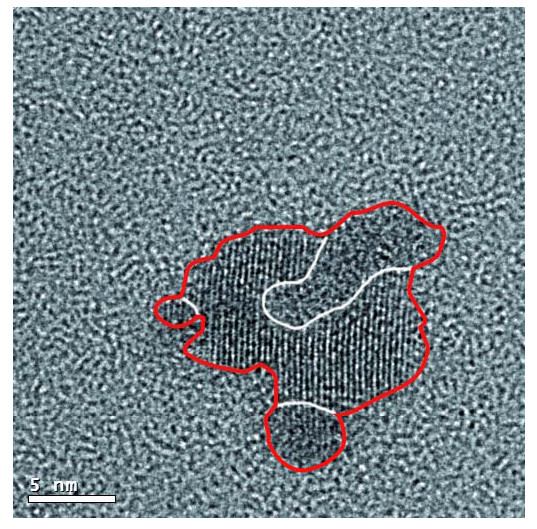
**HRTEM micrograph displays a nanoparticle (red contour) conformed by nanoparticles with different sizes (white contour)**.

Also, the figures display the corresponding size distributions (Figures 7b and 8b) and the nearest neighbor distances (Figures 7c and 8c). In Figure 7a, b, it can be seen that the nanoparticles have an average diameter of 5.7 nm and a distance between particles of 6.7 nm. When the bias voltage is increased at 6 kV, the average diameter and the *d*_NN _are enlarged to 6.7 nm and 22.9 nm, respectively. The increase of the nanoparticle size and the nearest neighbor distance when the bias voltage is raised can be explained by the coalescence of two or more nanoparticles, as shown in Figure 9. The bias polarization increases the cluster kinetic energy towards the substrate and enhances the nanoparticles' mobility on the surface, allowing that one nanoparticle could reach other nanoparticles.

### Quantum confinement effect of the reduced size of TiN nanoparticles

**Figure 10:**
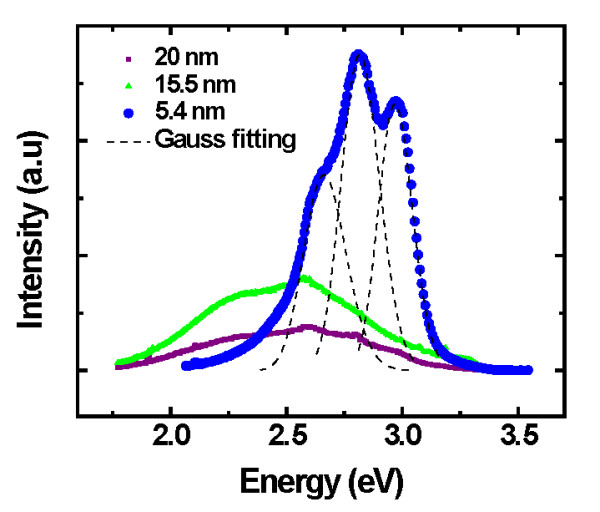
**Photoluminescence spectra of TiN nanoparticles with diameters of 20, 15.5, and 5.4 nm**.

Figure 10 displays the spectral PL of TiN nanoparticles at different sizes: 20, 15.5, and 5.4 nm. In the figure, the increase of the PL intensity when the diameter decreases from 20 to 15.5 nm can be observed. However, when the diameter decreases down to 5.4 nm, a strong visible PL is clearly seen. The origin of this strong PL is only due to the nanocluster size confinement because in transition metals, the light emission is normally very weak due to ultrafast nonradiative decay and the absence of a bandgap [21]. The PL has been previously reported in another transition metal nanoparticle with a size below 10 nm [22]. However, this is the first report in TiN nanoparticles.

**Figure 11:**
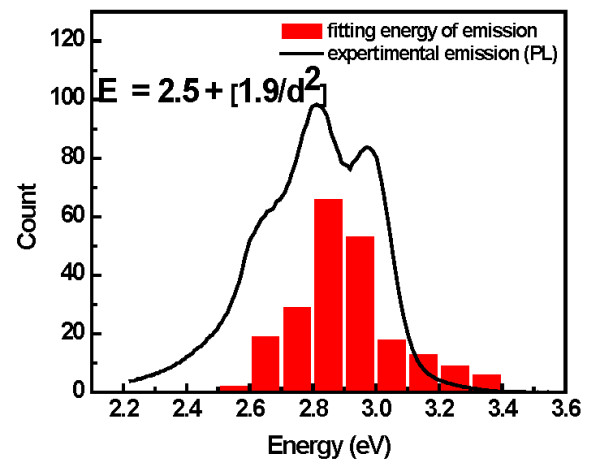
**Energy distribution by fitting equation of the PL spectrum**.

It can also be observed in Figure 10 that the strong PL is deconvoluted by three Gaussians, which correspond to the emission at different wavelengths. According to the bulk band structure studies, these emissions could be related to the transition between L1 and Gamma1. This is because the emission energies are close to the difference of energy band (2.3 ± 0.3 eV) [23]. In this case, we considered that the observation of three emissions is due to the size distribution on the surface (observed in AFM and HRTEM). A first attempt to explain this phenomenon is shown in Figure 11.

A fitting equation (Figure 11, inset) and the HRTEM diameter distribution of TiN nanoparticles at 3 kV were used to describe the PL intensity. It can be noticed that energy distribution, as a function of nanoparticle size, is in an agreement with PL peak position. The slight difference (2.5 eV) of both the theoretical energy band and the PL peaks are due to the quantum confinement effect, where the energy band is enlarged when the nanoparticle size becomes smaller (for spherical particles) [24,25]. Thus, in TiN, the PL peaks at a higher energy displacement with the nanoparticle size. For larger sizes, the energy displacement is less, (peaks or shoulders at approximately 2.6 eV) while for smaller sizes, the energy displacement is greater (peaks at approximately 3.0 eV).

## Conclusions

Size-selected TiN nanoparticles were produced on Si (111) substrates at room temperature by ICBD method. The critical parameters of the system were tuned to obtain small nanoparticles at different size: 4.1 ± 0.2, 5.3 ± 0.4, 6.2 ± 0.2, 7.1 ± 0.3, 14.9 ± 0.7, and 20.3 ± 0.8 nm. The nanoparticle size was controlled during the production process by a mass filter with a high resolution. After deposition, the size distribution was statistically analyzed using AFM images and was in excellent agreement with the filter mass.

The TiN nanoparticle size was directly measured by HRTEM micrograph, and the crystalline structure was confirmed by the FFT patterns. The nanoparticle shape was considered as quasi-spherical according to the AFM height profiles and plan-view TEM image. The size distribution, cover surface, and nearest neighbor distance were also statistically analyzed as a function of the bias voltage by HRTEM micrograph. The increase of nanoparticle size and the nearest neighbor distance when the bias voltage is raised were explained by the coalescence of two or more nanoparticles.

The PL spectra of TiN nanoparticles at different sizes were also investigated experimentally. An abrupt change in the optical properties is observed once the cluster diameter is 5.4 nm. The observation of three emissions in the strong luminescence was explained based on the quantum confinement effect due to the small size distribution of TiN nanoparticles on the surface. With this report, engineers and scientists may be able to produce small TiN nanoclusters and tailor the physical properties (hardness, optics, or electric) for useful applications (e.g., catalysis, electronics, and medicine).

## Competing interests

The authors declare that they have no competing interests.

## Authors' contributions

LCHM carried out the experiment and drafted the manuscript. JLM performed the AFM and HRTEM measurements. AFC and ER analyzed statistically the AFM and HRTEM images. GS carried out the measurement and interpretation of photoluminescence. EPT and LPC conceived the study, design, and coordination. All authors read and approved the final manuscript.
